# Strategies to prevent ventilator-associated pneumonia, ventilator-associated events, and nonventilator hospital-acquired pneumonia in acute-care hospitals: 2022 Update

**DOI:** 10.1017/ice.2022.88

**Published:** 2022-05-20

**Authors:** Michael Klompas, Richard Branson, Kelly Cawcutt, Matthew Crist, Eric C. Eichenwald, Linda R. Greene, Grace Lee, Lisa L. Maragakis, Krista Powell, Gregory P. Priebe, Kathleen Speck, Deborah S. Yokoe, Sean M. Berenholtz

**Affiliations:** 1 Department of Population Medicine, Harvard Medical School and Harvard Pilgrim Health Care Institute, Boston, Massachusetts; 2 Department of Medicine, Brigham and Women’s Hospital, Boston, Massachusetts; 3 Department of Surgery, University of Cincinnati Medicine, Cincinnati, Ohio; 4 Department of Medicine, University of Nebraska Medical Center, Omaha, Nebraska; 5 Division of Healthcare Quality Promotion, Centers for Disease Control and Prevention, Atlanta, Georgia; 6 Department of Pediatrics, Children’s Hospital of Philadelphia, Philadelphia, Pennsylvania; 7 Perelman School of Medicine, University of Pennsylvania, Philadelphia, Pennsylvania; 8 Highland Hospital, University of Rochester, Rochester, New York; 9 Stanford University School of Medicine, Palo Alto, California; 10 Department of Medicine, Johns Hopkins University School of Medicine, Baltimore, Maryland; 11 Department of Anesthesiology, Critical Care and Pain Medicine; Department of Pediatrics, Boston Children’s Hospital, Boston, Massachusetts; and Harvard Medical School, Boston, Massachusetts; 12 Department of Anesthesiology and Critical Care Medicine, The Johns Hopkins University School of Medicine, Baltimore, Maryland; 13 Department of Medicine, University of California San Francisco, San Francisco, California; 14 Department of Surgery, Johns Hopkins University School of Medicine, Baltimore, Maryland; 15 Department of Health Policy & Management, Bloomberg School of Public Health, Johns Hopkins University, Baltimore, Maryland

## Abstract

The purpose of this document is to highlight practical recommendations to assist acute care hospitals to prioritize and implement strategies to prevent ventilator-associated pneumonia (VAP), ventilator-associated events (VAE), and non-ventilator hospital-acquired pneumonia (NV-HAP) in adults, children, and neonates. This document updates the Strategies to Prevent Ventilator-Associated Pneumonia in Acute Care Hospitals published in 2014. This expert guidance document is sponsored by the Society for Healthcare Epidemiology (SHEA), and is the product of a collaborative effort led by SHEA, the Infectious Diseases Society of America, the American Hospital Association, the Association for Professionals in Infection Control and Epidemiology, and The Joint Commission, with major contributions from representatives of a number of organizations and societies with content expertise.

## Purpose

The purpose of this document is to highlight practical recommendations to assist acute-care hospitals to prioritize and implement strategies to prevent ventilator-associated pneumonia (VAP), ventilator-associated events (VAEs), and nonventilator hospitalacquired pneumonia (NV-HAP) in adults, children, and neonates. This document updates the *Strategies to Prevent Ventilator-Associated Pneumonia in Acute-Care Hospitals* published in 2014.^1^ This expert guidance document is sponsored by the Society for Healthcare Epidemiology (SHEA); it is the product of a collaborative effort led by SHEA, the Infectious Diseases Society of America, the Association for Professionals in Infection Control and Epidemiology, the American Hospital Association, and The Joint Commission, with major contributions from representatives of a number of organizations and societies with content expertise.

## Summary of major changes

This section lists major changes from the *Strategies to Prevent Ventilator-Associated Pneumonia in Acute-Care Hospitals*: 2014 Update^1^ including recommendations that have been added, removed, or altered. Recommendations are categorized as “essential practices” that should be adopted by all acute-care hospitals (in 2014 these were “basic practices,” renamed to highlight their importance as foundational for hospitals’ healthcare-associated infection (HAI) prevention programs) or as “additional approaches” that can be considered for use in locations and/or populations within hospitals when these HAIs are not controlled after implementation of essential practices (in 2014 these were “special approaches”). See Tables 2, 3, and 4 for a complete summary of the recommendations contained in this document.

### Essential practices

- Added a recommendation for high flow nasal oxygen or noninvasive positive pressure ventilation as options to avoid intubation, minimize duration of intubation, and prevent reintubations
- Added a recommendation for spontaneous awakening trials or sedation protocols as effective strategies to minimize sedation in adults
- Reclassified endotracheal tubes with subglottic secretion drainage from an Essential Practice to an Additional Approach
- Added a recommendation for daily toothbrushing
- Added a recommendation to use caffeine therapy to facilitate extubation in preterm neonates

### Additional approaches

- Reclassified endotracheal tubes with subglottic secretion drainage as an additional approach rather than an essential practice for adults and older children
- Added a recommendation to consider early tracheostomy
- Added a recommendation to consider postpyloric rather than gastric feeding in patients at high risk for aspiration

### Not recommended

- Oral care with chlorhexidine
- Probiotics
- Ultrathin polyurethane endotracheal tube cuffs
- Tapered endotracheal tube cuffs
- Automated control of endotracheal cuff pressures
- Frequent endotracheal cuff pressure monitoring

### New

- Section on prevention of nonventilator hospital-acquired pneumonia (NV-HAP)

## Intended use

This document was developed following the process outlined in the *Handbook for SHEA-Sponsored Guidelines and Expert Guidance Documents*.^2^ No guideline or expert guidance document can anticipate all clinical situations, and this document is not meant to be a substitute for individual clinical judgment by qualified professionals. This document is based on a synthesis of evidence, theoretical rationales, current practices, practical considerations, writing-group consensus, and consideration of potential harm, where applicable.

## Methods

SHEA recruited 2 subject-matter experts in the prevention of VAP, VAE, and NV-HAP to lead a panel of members representing the Compendium partnering organizations: SHEA, the Infectious Diseases Society of America (IDSA), the Association for Professionals in Infection Control and Epidemiology (APIC), the American Hospital Association (AHA), and The Joint Commission, as well as representation by the Centers for Disease Control and Prevention (CDC).

SHEA utilized a consultant medical librarian, who worked with each panel to develop a comprehensive search strategy for PubMed and Embase (January 2012–July 2019; updated to August 2021). Articles’ abstracts were reviewed by panel members in a double-blind fashion using the abstract management software Covidence (Melbourne, Australia), and potentially relevant articles were reviewed as full text. The Compendium Lead Authors subsequently voted to update the literature findings, and the librarian reran the search to include articles published through August 2021. Panel members reviewed the abstracts of these articles via Covidence and incorporated relevant references.

Recommendations resulting from this literature review process were classified based on the quality of evidence and the balance between desirable and potentially undesirable effects of various interventions (see Table 1). Panel members met via video conference to discuss literature findings; recommendations; quality of evidence for these recommendations; and classification as essential practices, additional approaches, or unresolved issues. Panel members reviewed and approved the document and its recommendations.

The Compendium Expert Panel, made up of members with broad healthcare epidemiology and infection prevention expertise, reviewed the draft manuscript after consensus had been reached by writing panel members.

Following review and approval by the Expert Panel, the 5 partnering organizations, stakeholder organizations, and the CDC reviewed the document. Prior to dissemination, the guidance document was reviewed and approved by the SHEA Guidelines Committee, the IDSA Standards and Practice Guidelines Committee, and the Boards of SHEA, IDSA, APIC, AHA, and The Joint Commission.

All panel members complied with SHEA and IDSA policies on conflict-of-interest disclosure.

## Section 1: Rationale and statements of concern

1. Hospitalized patients are at high risk for pneumonia and other pulmonary complications, particularly patients on mechanical ventilation.
  - Hospital-acquired pneumonia is the most common nosocomial infection.^3^ It affects ~1 in 100 patients overall and up to 1 in 10 patients on invasive mechanical ventilation.^3,4^ The true incidence of nosocomial pneumonia is difficult to discern, however, because diagnostic criteria vary widely, correlate poorly with histology, are often subjective, and are applied differently by different surveyors.^5–8^
  - Many hospitals have reported dramatic decreases in VAP rates over the past 20 years, but the extent to which these declines reflect better care versus stricter application of subjective surveillance criteria remains unclear.^9,10^ Clinical surveys suggest that 5%–10% of ventilated patients continue to be treated for VAP,^11–14^ and an independent audit conducted by the Centers for Medicare and Medicaid Services reported that VAP rates were stable between 2005 and 2013.^15^
  - Patients on mechanical ventilation are at risk for a variety of serious complications in addition to pneumonia. Examples include acute respiratory distress syndrome, fluid overload, atelectasis, pneumothorax, barotrauma, and pulmonary embolism. The CDC created VAE definitions to better capture the breadth of serious complications associated with mechanical ventilation.^16^ Approximately 5%–10% of mechanically ventilated patients develop VAEs.^17–24^
  - The coronavirus disease 2019 (COVID-19) pandemic has been associated with an increase in the incidence of nosocomial pneumonia due to hospital-acquired severe acute respiratory coronavirus virus 2 (SARS-CoV-2) infections and hospital-acquired bacterial superinfections in patients admitted with COVID-19 pneumonia.^25–27^ Differentiating superinfection from underlying COVID-19 pneumonia and COVID-19–related ARDS is challenging due to considerable overlap in clinical signs.
2. VAP, VAE, and NV-HAP are detrimental to patients and increase costs.
  - The attributable mortality of VAP is estimated to be ~10% but varies considerably by type and severity of underlying illness.^28–32^
  - VAP and VAE extend duration of invasive mechanical ventilation, prolong intensive care unit (ICU) and hospital length of stay, and increase mortality risk.^18,21–23,31,33,34^ They are also associated with greater use of antimicrobials and higher costs.^19,33^ Patients with VAEs are ~50% more likely to die compared to similar patients with VAP.^35^ NV-HAP is associated with a doubling of the length of time until discharge and substantially higher mortality rates compared to similar patients without NV-HAP.^36,37^

## Section 2: Background on detection of VAP, VAE, and NV-HAP

1. Despite pneumonia’s clinical importance, our ability to conduct accurate pneumonia surveillance is very limited.
  - Pneumonia is usually defined by clinical, radiographic, and microbiological criteria. These signs are neither sensitive nor specific relative to histopathology.^6,38–40^ In addition, the signs and symptoms used to diagnose pneumonia are subjective, which leads to substantial interobserver variability.^8,12,13,41–43^ Administrative data are similarly inaccurate.^11,44–47^ Improvements in VAP rates do not reliably correlate with improvements in outcomes.^48,49^
    - The weaknesses of traditional pneumonia surveillance definitions limit their utility for measuring the impact of care improvement programs and for benchmarking quality of care between different healthcare facilities.^50–53^
  - CDC’s VAE framework:
  - The CDC created VAE definitions to try to overcome the subjectivity, complexity, and limited focus of traditional VAP surveillance definitions.^16^ VAE definitions were designed to capture a range of severe complications of mechanical ventilation while being objective, reproducible, and amenable to automation.
  - VAEs are triggered by sustained increases in ventilator settings after a period of stable or decreasing ventilator settings.
  - There are 3 nested tiers of VAEs in adults: ventilator-associated conditions (VACs), infection-related ventilatorassociated complications (IVACs), and possible VAP (PVAP). Similar subcategories for pediatric patients are being evaluated but have not yet been adopted by the CDC.^54,55^
    - In adults, VAEs and VACs are synonymous and are defined as an increase in the daily minimum positive end expiratory pressure (PEEP) of ≥3 cm H_2_O sustained for ≥2 calendar days after ≥2 days of stable or decreasing daily minimum PEEP, or an increase in the fraction of inspired oxygen (FiO_2_) of ≥20 points sustained for ≥2 days after ≥2 days of stable or decreasing daily minimum FiO_2_ levels.^56^
    - IVAC is defined as a VAC with concurrent indications of possible infection, namely an abnormal temperature (<36 or >38°C) or white blood cell count (≤4,000 or ≥12,000 cells/mm^3^), and 1 or more new antibiotic starts that continue for ≥4 days, all beginning within 2 days before or 2 days after VAC onset.^56^
    - PVAP is defined as an IVAC with indications that infection might be localized to the lungs. It requires respiratory secretion cultures positive for potentially pathogenic organisms, positive cultures from pleural fluid, positive assays for respiratory viruses or *Legionella*, or suggestive histopathology concurrent with the IVAC. The culture criterion can be fulfilled via quantitative cultures above various thresholds that vary depending upon specimen type or through positive cultures with any amount of growth if there is concurrent Gram-stain evidence of purulence.^56^
  - In children and neonates, a pediatric VAE (PedVAE) is defined as an increase in the daily minimum mean airway pressure of ≥4 cm H_2_O sustained for ≥2 calendar days after ≥2 days of stable or decreasing daily minimum mean airway pressure, or an increase in FiO_2_ of ≥25 points sustained for ≥2 days after ≥2 days of stable or decreasing daily minimum FiO_2_s.^54^
  - There are 2 subcategories of VAEs in adults: infection-related ventilator-associated complications (IVAC) and possible VAP (PVAP). Similar subcategories for pediatric patients are being evaluated but have not yet been adopted by the CDC.^55,57^
  - VAE is not synonymous with VAP. Only ~25%–33% of VAEs are due to pneumonia, and many mild pneumonias do not meet the VAE thresholds for increased ventilator settings.^35^
  - Qualitative studies suggest that most VAEs in adults and children are caused by pneumonia, fluid overload, atelectasis, and/or ARDS.^17,19,22,58–63^
  - Potential risk factors for VAE include sedatives (especially benzodiazepines and propofol),^64–67^ opioids,^64^ positive fluid balance,^60,63,64,66,68–71^ mandatory modes of mechanical ventilation with high tidal volumes and/or high inspiratory driving pressures,^60,64,69,72^ blood transfusions,^63,68,73^ oral care with chlorhexidine,^74^ stress ulcer prophylaxis,^75^ patient transport,^76^ gastric retention,^66^ reintubation,^68^ and neuromuscular blockade.^63,64,69^ Dexmedetomidine, spontaneous awakening and breathing trials, and conservative fluid management may be protective.^21,65,75,77,78^
  - A growing body of literature demonstrates the preventability of VAEs.^21,77–83^ The best-studied interventions to date that have been associated with lower VAE rates in interventional trials include spontaneous awakening trials, spontaneous breathing trials, and conservative fluid management.^21,77–79,81,82^
2. Recommended surveillance strategies:
  - The CDC recommends that hospitals conduct surveillance for VAE in lieu of VAP using CDC definitions and surveillance protocols.^56^
  - VAE definitions are amenable to partial or complete automation using electronic data.^22,84–87^ Facilities seeking to automate VAE detection should work with their information technology personnel and/or electronic health record vendor(s).
  - Alternatively, infection preventionists should work with their critical care, respiratory therapy, and/or information technology staff to develop efficient means to gather and aggregate ventilator data (daily minimum PEEP and daily minimum FiO_2_) from all patients ventilated for ≥4 days.
    - Temperature, white blood cell count, and antibiotic exposure data are only needed for the subset of patients with VAEs to determine whether they fulfill IVAC criteria. Pulmonary-specimen Gram stains and microbiology test results are only required for the subset of patients who meet IVAC criteria to determine whether they fulfill PVAP criteria.
    - Organizing daily ventilator data into ‘line lists’ for every patient with 1 row of data per patient per calendar day facilitates VAE detection by allowing the surveyor to vertically scan daily ventilator settings to look for sustained increases that cross the threshold for VAE.^88^ Surveyors can also enter their data into the CDC online VAE calculators to assist with case identification (adult VAE: http://www.cdc.gov/nhsn/VAE-calculator/index.html; pediatric VAE (PedVAE): https://www.cdc.gov/nhsn/pedvae-calculator/index.html).
  - The CDC has not yet developed NV-HAP surveillance definitions. The CDC is exploring the feasibility, reliability, and significance of low-burden reporting options that utilize readily available electronic data.^36,89^

## Section 3: Background on prevention of VAP, VAE, and NV-HAP

### Framework for evaluating and prioritizing interventions

1. The subjectivity and lack of specificity of diagnosing pneumonia complicate the interpretation of VAP and NV-HAP prevention studies.^90^ Subjectivity makes it possible that decreases in observed pneumonia rates are due to stricter interpretation of subjective diagnostic criteria rather than true decreases in disease. Lack of specificity makes it possible that lower pneumonia rates are due to less colonization or decreases in conditions that mimic the presentation of pneumonia without corresponding decreases in true pneumonia cases.
2. VAE criteria are more objective and hence less susceptible to these sources of bias, but the literature on VAE prevention is still relatively sparse.
3. Given the limitations of the pneumonia prevention literature and the relative paucity of VAE prevention literature, we prioritize pneumonia prevention strategies associated with improvements in objective outcomes such as duration of mechanical ventilation, ICU or hospital length of stay, mortality, VAEs, antibiotic utilization, and/or costs in randomized controlled trials. In addition, the potential benefits of different interventions are balanced against their feasibility, costs, and potential harms.

## Section 4: Recommended strategies to prevent VAP, VAE, and NV-HAP

Recommendations are classified as either: 1) essential practices that improve objective outcomes such as duration of mechanical ventilation, length of stay, mortality, VAEs, antibiotic utilization, and/or costs with little risk of harm that should be adopted by all hospitals. We also recommend interventions that are outcome-neutral but cost saving. Or 2) additional approaches that improve objective outcomes (including VAE) but carry some risk of harm, and interventions that lower VAP or NV-HAP rates, but where insufficient data exist to determine their impact on objective outcomes. Hospitals can consider adopting additional approaches if their VAE, VAP, or NV-HAP rates do not improve despite high performance rates of essential practices. Interventions that do not improve VAE, VAP, or NV-HAP rates nor objective outcomes are not recommended. The quality of evidence rating scheme is summarized in Table 1. Recommended strategies are summarized in Table 2 for adults, Table 3 neonates, and Table 4 for pediatric patients.

### Essential practices for preventing VAP and/or VAEs in adult patients

Interventions with little risk of harm that are associated with decreases in duration of mechanical ventilation, length of stay, mtality, antibiotic utilization, and/or costs.

#### Avoid intubation and reintubation if possible

1. **Use high-flow nasal oxygen or non-invasive positive pressure ventilation (NIPPV) as appropriate whenever safe and feasible** (Quality of Evidence: HIGH).
  - High-flow nasal oxygen may help avert intubation in patients with hypoxemic respiratory failure and prevent reintubation after extubation of critically ill patients and postoperative patients compared to conventional oxygen therapy.^91–94^ High-flow nasal oxygen has also been associated with a trend toward less nosocomial pneumonia in patients with hypoxemic respiratory failure.^95^
  - NIPPV is associated with lower rates of intubation, reintubation, VAP, and mortality compared to conventional oxygen therapy in patients with acute hypercapnic or hypoxemic respiratory failure.^96–100^ Use caution when considering NIPPV to manage patients with impaired consciousness, acute lung injury, acute respiratory distress syndrome, severe hypoxemia, severe acidemia, or when continuing NIPPV for patients whose dyspnea or gas exchange fails to rapidly respond to NIPPV. Helmet ventilation may be associated with better outcomes than facemask ventilation.^99,101^
  - High-flow nasal oxygen and NIPPV appear to be similar in their capacity to prevent intubation, reintubation, and postextubation respiratory failure. Some meta-analyses suggest that high-flow nasal cannula may reduce ICU and hospital length of stay compared to NIPPV, while others do not.^92,102^
  - Combining high-flow nasal oxygen with NIPPV immediately after extubation may further decrease the risk of reintubation in patients at high risk for extubation failure compared to using high-flow nasal oxygen alone.^103^
2. **Placing nonintubated patients with COVID-19 acute hypoxemic respiratory failure in the prone position may lower the risk of intubation compared to standard care** (Quality of Evidence: MODERATE).^104^

#### Minimize sedation

1. **Minimize sedation of ventilated patients whenever possible** (Quality of Evidence: HIGH).^105,106^
2. **Preferentially use multimodal strategies and medications other than benzodiazepines to manage agitation** (Quality of Evidence: HIGH).^106^
  - Examples include analgesics for pain, reassurance for anxiety, and antipsychotics, dexmedetomidine, and/or propofol for agitation.^106^ Dexmedetomidine and propofol are associated with shorter duration of mechanical ventilation and ICU length of stay compared to benzodiazepines.^107^ A randomized trial of light sedation with dexmedetomidine versus propofol found no difference in ventilator-free days or mortality.^108^ Dexmedetomidine may decrease need for intubation in patients on noninvasive ventilation.^109^
3. **Utilize a protocol to minimize sedation** (Quality of Evidence: HIGH).^110^
  - Potential strategies to minimize sedation include nurse-driven protocols for targeted light sedation and daily sedative interruptions (ie, spontaneous awakening trials) for patients without contraindications.^106,110^
  - A meta-analysis of 6 randomized trials reported that protocols to minimize sedation were associated with significantly shorter ICU length of stay compared to managing patients without protocols.^110^ There was no significant association between the use of protocols to minimize sedation and duration of mechanical ventilation or short-term mortality. There was insufficient evidence to recommend one protocol over another.
  - A small, single-center, randomized trial^111^ suggested that patients managed with no sedation (but morphine as needed) versus propofol or midazolam may be extubated sooner and have shorter ICU length of stay, but a subsequent multicenter randomized trial of no sedation versus light sedation with daily sedative interruptions reported no difference in ventilator-free days, ICU-free days, or 90-day mortality.^112^
4. **Implement a ventilator liberation protocol** (Quality of Evidence: HIGH)^113^
  - Assess readiness to extubate daily in patients without contraindications (i.e., conduct spontaneous breathing trials).^114–117^
  - Ventilator liberation protocols are associated with extubating patients an average of 1 day earlier compared to managing patients without a protocol.^113^
  - Protocols to minimize sedation, mobilize patients, andliberate them from mechanical ventilation may be synergistic.^118,119^

#### Maintain and improve physical conditioning

1. **Provide early exercise and mobilization** (Quality of Evidence: MODERATE).
  - Early exercise and mobilization programs may shorten duration of mechanical ventilation, reduce ICU length of stay, lower VAP rates, and increase the rate of return to independent function.^113,120–123^ There is no consistent association between early mobilization and hospital length of stay or mortality.
  - Financial modeling suggests that early mobility programs may be cost saving.^119,124^

#### Elevate the head of the bed

1. **Elevate the head of the bed to 30–45°** (Quality of Evidence: LOW).
  - A meta-analysis of 8 randomized trials reported that elevating the head of the bed was associated with a significant reduction in VAP rates but no difference in duration of mechanical ventilation or mortality.^125^ The data on outcomes other than VAP, however, were sparse (combined sample size <500 patients); thus, the impact on these outcomes is uncertain. Given the simplicity, ubiquity, minimal risk, lack of cost, and potential benefit of this intervention, we nonetheless classify it as an essential practice while we await further data.

#### Provide oral care with toothbrushing but without chlorhexidine

1. **Provide daily oral care with toothbrushing but without chlorhexidine** (Quality of Evidence: MODERATE).
  - Daily toothbrushing is associated with significantly lower VAP rates, shorter duration of mechanical ventilation, and shorter ICU length of stay.^126,127^
  - Meta-analyses of randomized trials and observational studies allow for the possibility that oral care with chlorhexidine may increase mortality rates.^75,128–130^ This is further discussed below.

#### Provide early enteral rather than parenteral nutrition

1. **Provide early enteral rather than parenteral nutrition** (Quality of Evidence: HIGH).
  - Early enteral nutrition is associated with a lower risk of nosocomial pneumonia, shorter ICU length of stay, and shorter hospital length of stay compared to early parenteral nutrition.^131^

#### Maintain ventilator circuits

1. **Change the ventilator circuit only if visibly soiled or malfunctioning** (Quality of Evidence: HIGH).
  - Changing the ventilator circuit as needed rather than on a fixed schedule has no impact on VAP rates or patient outcomes but decreases costs.^132^
  - Follow manufacturers’ instructions for use if they differ from this recommendation.
  - Follow CDC/Healthcare Infection Control Practices Advisory Committee guidelines and manufacturers’ instructions for use of sterilization and disinfection of respiratory care equipment.^133^

### Additional approaches for preventing VAP and/or VAEs in adult patients

Additional approaches are interventions associated with lower VAP rates that that may also decrease VAE rates, duration of mechanical ventilation, length of stay, and/or mortality but carry some risk of harm. Additional approaches also include interventions that are associated with lower VAP rates, but insufficient data exist to determine their impact on objective outcomes. Hospitals can consider adopting additional approaches if their VAP or VAE rates do not improve despite high performance rates with essential practices.

The following interventions may decrease duration of mechanical ventilation, length of stay, and/or mortality in some populations but not in others, and they may confer some risk of harm in some populations.

1. **Consider using selective decontamination of the oropharynx and digestive tract to decrease microbial burden in ICUs with low prevalence of antibiotic-resistant organisms.**^128,134,135^ **Antimicrobial decontamination is not recommended in countries, regions, or ICUs with high prevalence of antibiotic-resistant organisms**^136^ (Quality of Evidence: HIGH).
  - A meta-analysis of 6 cluster randomized trials performed in countries with low levels of antibiotic resistance reported that selective decontamination of the oropharynx with topical antibiotics was associated with a 16% reduction in hospital mortality, and decontamination of the oropharynx and digestive tract with a combination of topical, oral, and parenteral antibiotics was associated with an 18% reduction in hospital mortality.^135^ Selective digestive decontamination was more effective than selective oral decontamination alone (OR, 0.90; 95% CI, 0.82–0.97 for hospital death).^135^ A broader meta-analysis that included a larger but more heterogenous set of studies had similar findings.^137^
    - Oral agents that have been used for digestive decontamination include colistin, tobramycin, and amphotericin B. Parenteral agents include cefotaxime.
    - ICUs that implement this practice should actively monitor its impact on antibiotic utilization, antimicrobial resistance, and *Clostridioides difficile* infections.
    - There is no consensus on what constitutes “low levels of antibiotic resistance,” but an arbitrary threshold that has been used by other guidelines and randomized trials is <5% of bloodstream infections caused by extended-spectrum β-lactamase–producing Enterobacterales.^136,138^
  - A cluster randomized trial of selective digestive decontamination (without parenteral antibiotics) versus selective oral decontamination versus oral care with 2% chlorhexidine versus routine care conducted in ICUs with high levels of antibiotic resistance (≥5% of bloodstream infections caused by extended-spectrum β-lactamase–producing Enterobacterales) found no difference between study arms in ICU-acquired bloodstream infections or 28-day mortality rates.^136^ Selective oral and digestive decontamination with antibiotics is therefore not recommended in settings with high baseline levels of antibiotic resistance.

The following interventions may lower VAP rates, but current data are insufficient to determine their impact on duration of mechanical ventilation, length of stay, and mortality.

1. **Consider using endotracheal tubes with subglottic secretion drainage ports to minimize pooling of secretions above the endotracheal cuff in patients likely to require >48–72 hours of intubation** (Quality of Evidence: MODERATE).
  - Intermittent and continuous drainage of subglottic secretions has been studied in at least 20 randomized controlled trials. On meta-analysis, the use of endotracheal tubes with subglottic drainage reduced VAP rates by 44%.^139^ There was no association, however, between subglottic secretion drainage and duration of mechanical ventilation, ICU length of stay, or hospital length of stay. One meta-analysis reported a significant impact on mortality but abstracted one large study twice.^139,140^ The effect on mortality was no longer significant after removing the duplicate study (OR, 0.92; 95% CI, 0.83–1.02). One large trial included VAE as an outcome and found no association between subglottic secretion drainage and VAE rates.^140^ Some studies have reported that subglottic secretion drainage is associated with less antibiotic utilization, but others have not.^140–142^
  - Reductions in duration of mechanical ventilation with subglottic secretion drainage appear to be limited to patients expected to require >48–72 hours of mechanical ventilation.^143^ Endotracheal tubes with subglottic secretion drainage ports should therefore be reserved for patients likely to require >48–72 hours of intubation. Patients requiring emergency intubation in the hospital and preoperative patients at risk for prolonged mechanical ventilation are reasonable candidates.
  - Extubation followed by immediate reintubation to exchange a conventional endotracheal tube for a subglottic secretion drainage endotracheal tube is not recommended.
2. **Consider early tracheostomy** (Quality of Evidence: MODERATE).
  - Meta-analysis of 17 randomized trials suggests that early tracheostomy (within 7 days of intubation) may be associated with a 40% decrease in VAP rates, less time on mechanical ventilation, and fewer ICU days but no difference in mortality.^144^
  - Decision makers should integrate these potential benefits with each patient’s values and preferences when determining whether and when to proceed with tracheostomy.^145^
3. **Consider postpyloric feeding tube placement in patients with gastric feeding intolerance at high risk for aspiration** (Quality of Evidence: MODERATE).
  - Postpyloric feeding is associated with less aspiration and less pneumonia compared to gastric-tube feeding. Meta-analyses vary in their assessment of whether postpyloric feeding is associated with decreases in ventilator, ICU, and/or hospital length of stay.^146,147^
  - Postpyloric tube placement requires special expertise that is not available in all centers and may incur delay in placement. Postpyloric feeding is considered less physiologic than gastric feeding.^131^
  - Postpyloric feeding should therefore be reserved for patients with gastric feeding intolerance and for patients at high risk for aspiration as detailed in nutrition society guidelines.^131,148,149^

### Approaches that should not be considered a routine part of VAP and/or VAE prevention in adult patients

The following interventions are inconsistently associated with lower VAP rates and have no impact or negative impact on duration of mechanical ventilation, length of stay, or mortality.

1. **Oral care with chlorhexidine** (Quality of Evidence: MODERATE)
  - Oral care with chlorhexidine has been studied in multiple randomized controlled trials.^150^ The impact of oral care with chlorhexidine on pneumonia rates is unclear. Meta-analyses report significantly lower VAP rates, but this signal is driven by unblinded studies. There was no association between oral care with chlorhexidine and lower VAP rates when the analysis was restricted to double-blinded studies.^129^ Meta-analyses of both blinded and unblinded studies also show no impact on duration of mechanical ventilation or ICU length of stay.^129,150^ Chlorhexidine’s lack of impact on VAP, duration of mechanical ventilation, or ICU length of stay was echoed in a large randomized trial of chlorhexidine de-adoption versus usual care.^151^
  - Some meta-analyses of randomized trials and some observational studies report an association between oral care with chlorhexidine and higher mortality rates.^75,128–130^ The mortality signal is uncertain, however, because other meta-analyses did not find higher mortality rates, there was no change in mortality observed in a large randomized trial of chlorhexidine de-adoption, and the observational studies may be at risk of residual confounding.^126,151,152^ Nonetheless, given chlorhexidine’s lack of clear impact on VAP rates and the possibility of harm, routine oral care with chlorhexidine is not recommended. Oral care including toothbrushing without chlorhexidine, however, is considered an essential practice.
2. **Probiotics** (Quality of Evidence: MODERATE)
  - Multiple meta-analyses of randomized controlled trials have reported a possible association between probiotics and lower VAP rates, but these analyses have included many studies at high risk of bias due to lack of blinding.^153–156^ There is no association between probiotics and VAP when restricting the analysis to double-blinded studies.^155^ This finding was mirrored in a large, rigorous, multicenter, randomized trial conducted after the most recent meta-analysis.^157^ Neither this trial nor the meta-analyses found a significant impact on ICU length of stay, hospital length of stay, or mortality.
  - Probiotics should not be used in patients with compromised immune systems or gastrointestinal diseases that increase the risk of gut translocation. Multiple cases of fungemia or bacteremia have been reported in patients administered probiotics as have cases of aerosol transmission of probiotics within ICUs.^158–164^
3. **Ultrathin polyurethane endotracheal tube cuffs** (Quality of Evidence: MODERATE)
  - Ultrathin polyurethane cuffs seal more uniformly against the tracheal wall and may therefore allow fewer secretions to seep around the cuff and into the lungs. Two small randomized trials^165,166^ reported lower VAP rates but a larger, more rigorous study found no difference in VAP rates, duration of endotracheal intubation, or ICU length of stay.^167^ Similarly, there were no significant associations between polyurethane cuffs and VAP rates, duration of mechanical ventilation, ICU length of stay, or mortality on meta-analysis.^168^
4. **Tapered endotracheal tube cuffs** (Quality of Evidence: MODERATE)
  - A meta-analysis of 5 randomized trials of tapered versus conical endotracheal tube cuffs found no differences in VAP rates, duration of mechanical ventilation, ICU length of stay, hospital length of stay or mortality.^169^
5. **Automated control of endotracheal-tube cuff pressure** (Quality of Evidence: MODERATE)
  - Automated control of endotracheal-tube cuff pressure was associated with lower VAP rates in 2 small trials but this signal has not been borne out in other trials.^170–174^ Indeed, 2 large, multicenter, randomized trials of automated cuff-pressure regulation versus thrice-daily manual cuff-pressure assessments found no difference between arms in VAP, VAE, antibiotic utilization, duration of mechanical ventilation, ICU length of stay, or mortality.^174,175^
6. **Frequent endotracheal-tube cuff-pressure monitoring** (Quality of Evidence: MODERATE)
  - A single-center prospective trial found no advantage to more frequent versus less frequent cuff-pressure monitoring.^176^ At least 1 laboratory investigation suggests that manual measurement of cuff pressure is associated with loss of cuff pressure and potential leakage of fluid around the cuff.^177^
7. **Silver-coated endotracheal tubes** (Quality of Evidence: MODERATE)
  - A large, multicenter, randomized controlled trial found that silver-coated endotracheal tubes reduced VAP rates by 36%. However, the organisms associated with VAP included nonpathogenic colonizers, and there was no impact on mean duration of mechanical ventilation, hospital length of stay, or mortality.^178,179^
8. **Kinetic beds** (continuous lateral rotational therapy and oscillation therapy) (Quality of Evidence: MODERATE)
  - A meta-analysis of 15 randomized controlled trials found a significant decrease in VAP rates but no impact on duration of mechanical ventilation or mortality.^180^ The meta-analysis researchers warned that the observed reduction in VAP rates might be artifactual given weaknesses in contributing studies’ design and execution.
9. **Prone positioning** (Quality of Evidence: MODERATE)
  - Prone positioning is associated with lower mortality rates among patients with moderate-to-severe ARDS, but this signal appears to be independent of VAP. Prone positioning may be indicated for reasons other than VAP prevention.^181–183^
10. **Chlorhexidine bathing** (Quality of Evidence: MODERATE)
  - Observational studies have suggested that chlorhexidine bathing may reduce the risk of VAP, but this finding has not been borne out in randomized trials.^184–186^ Chlorhexidine bathing is, however, beneficial in preventing other healthcare-associated infections.^187^

#### Approaches that definitively are not recommended for VAP or VAE prevention

Good-quality evidence suggests that the following interventions neither lower VAP/VAE rates nor decrease duration of mechanical ventilation, length of stay, or mortality.

1. **Stress-ulcer prophylaxis** (Quality of Evidence: MODERATE)
  - Stress-ulcer prophylaxis lowers the risk of gastrointestinal bleeding, but a meta-analyses of randomized trials suggested no impact on nosocomial pneumonia, length of stay, or mortality.^188–192^ A large, multicenter randomized trial of pantoprazole versus placebo in ICU patients reported no difference in pneumonia rates or mortality rates.^193^
  - Stress-ulcer prophylaxis may be indicated for reasons other than VAP prevention.
2. **Monitoring residual gastric volumes** (Quality of Evidence: MODERATE)
  - Monitoring patients for regurgitation and vomiting alone is as effective as monitoring patients for regurgitation, vomiting, and residual gastric volumes with regard to VAP rates, duration of mechanical ventilation, and mortality.^194^
3. **Early versus late parenteral nutrition** (Quality of Evidence: MODERATE)
  - Early parenteral nutrition (within 48 hours of ICU admission) is associated with increased mortality and nosocomial infections compared to late parenteral nutrition (initiated on or after ICU day 8).^195^

### Approaches that are neither recommended nor discouraged for VAP prevention in adult patients

These interventions have no impact on VAP rates or patient outcomes and have unclear impact on costs.

1. **Closed endotracheal tube suctioning systems** (Quality of Evidence: MODERATE)
  - Meta-analyses have found no difference in VAP rates, duration of mechanical ventilation, ICU length of stay or mortality between patients randomized to open versus closed endotracheal suctioning systems.^196–198^ A crossover trial in 4 ICUs found no difference between open versus closed systems in patient-to-patient transmissions of gram-negative pathogens.^199^ Different trials have reached different conclusions regarding cost.^197,200,201^

#### Preventing VAP and/or VAEs in neonatal patients

1. Framework for evaluating and prioritizing interventions:
  - Very few studies in neonates have evaluated the impact of VAP or VAE prevention interventions on duration of mechanical ventilation, length of stay, or mortality; therefore, we evaluated potential interventions on the basis of safety, feasibility, and potential impact on VAP and PedVAE rates. Interventions that lower VAP or PedVAE rates and confer minimal risks of harm are classified as essential practices. Interventions with unproven but potential impact on VAP or PedVAE rates and minimal risk of harm are classified as additional approaches. Hospitals can consider additional approaches if their VAP or PedVAE rates do not improve despite high performance rates for essential practices. Interventions with unknown benefits, known risks of harm, or unknown risks of harm are not recommended.

### Special considerations in preterm neonates

1. Clinical signs used to diagnose VAP and VAE in adults have limited utility in preterm neonates. Fever rarely occurs in preterm neonates because they are prone to hypothermia and are therefore often thermoregulated with heated incubators or radiant warmers. Worsening gas exchange or apnea can be caused by significant nonpulmonary illnesses, including sepsis and necrotizing enterocolitis. New or progressive infiltrates in ventilated preterm neonates often indicate progression of chronic lung disease rather than new infection.
2. Adult VAE definitions are not suitable for neonates because they do not reflect standard ventilator management practices for this population. The CDC recently published VAE definitions for children and neonates, denoted PedVAE, based on sustained increases in daily minimum mean airway pressure and/or FiO_2_ but data on the incidence, causes, and preventability of PedVAE in preterm infants are sparse.^54,55,63,202^
3. Pooled mean VAP rates for neonates reported to CDC’s National Healthcare Safety Network (NHSN) in 2011 ranged from 0.2 to 1.8 infections per 1,000 ventilator days.^203^ Whether these rates are broadly representative of all neonatal units remains unknown, however, because many hospitals do not perform VAP surveillance for neonates (especially those born preterm) in light of the limitations of VAP definitions. VAP rates in NICUs are no longer reported to the NHSN.

### Essential practices for preterm neonates

These interventions confer minimal risk of harm and may lower VAP and/or PedVAE rates.

#### Avoid intubation

1. **Avoid intubation if possible** (Quality of Evidence: HIGH).
  - Nasal continuous positive airway pressure (CPAP) ventilation (with or without nasal intermittent mechanical ventilation) and high-flow oxygen via nasal cannula are viable alternatives to intubation in most preterm infants, but success rates are greatest for those delivered at >28 weeks gestation.^204–208^
  - Many premature neonates (especially those with a gestational age >28 weeks) can be successfully supported with noninvasive positive pressure ventilation in the delivery room and subsequently in the NICU.

#### Minimize duration of mechanical ventilation

1. **Manage patients without sedation whenever possible**^209,210^ (Quality of Evidence: LOW).
2. **Use caffeine therapy for apnea of prematurity within 72 hours after birth to facilitate extubation**^211^ (Quality of Evidence: HIGH).
3. **Assess readiness to extubate daily** (Quality of Evidence: LOW).
4. **Take steps to minimize unplanned extubations and reintubations**^212,213^ (Quality of Evidence: LOW).
5. Use nasal CPAP or nasal NIPPV in the postextubation period to help prevent the need for reintubation.^214^
6. **Provide regular oral care with sterile water (extrapolated from practice in infants and children, no data in preterm neonates**) (Quality of Evidence: LOW).
7. **Change the ventilator circuit only if visibly soiled or malfunctioning or per manufacturers’ instructions for use (extrapolated from studies in adults and children, no data in preterm neonates)** (Quality of Evidence: LOW).

### Additional approaches for preterm neonates

These interventions have minimal risks of harm, but their impact on VAE and VAP rates is unknown.

1. **Lateral recumbent positioning**^215^ (Quality of Evidence: LOW)
2. **Reverse Trendelenburg positioning** (Quality of Evidence: LOW)
3. **Closed/in-line suctioning**^216,217^ (Quality of Evidence: LOW)
4. **Oral care with maternal colostrum**^218^ (Quality of Evidence: MODERATE)

### Approaches that are generally not recommended for preterm neonates

This intervention has inadequate data on risks and unknown impact on VAP rates in preterm neonates.

1. **Regular oral care with an antiseptic or Biotene**^219^ (Quality of Evidence: LOW).
  - Data are insufficient regarding the impact of altering neonatal microflora and whether oral antiseptics are absorbed across the oral mucosa of preterm neonates.

These interventions may be harmful to preterm neonates:

1. **Histamine H2-receptor antagonists** (Quality of Evidence: MODERATE)
  - H2-receptor antagonists may increase the risk of nosocomial infection and mortality in preterm neonates.^220,221^
2. **Prophylactic broad-spectrum antibiotics** (Quality of Evidence: MODERATE)
  - Prophylactic broad-spectrum antibiotics are associated with increased risk of necrotizing enterocolitis, prolonged length of stay, and death in premature infants.^222–225^
3. **Spontaneous breathing trials** (Quality of Evidence: LOW)
  - Ventilating preterm neonates with prolonged continuous positive airway pressure alone increases the risk of extubation failure.^226,227^

### Approaches that are not applicable to preterm neonates

1. **Daily interruption of sedation** (Quality of Evidence: LOW)
  - Sedation is not routinely used for neonates on mechanical ventilation.
2. **Prophylactic probiotics and synbiotics** (Quality of Evidence: LOW)
  - Currently, no products have been approved by the FDA for preterm neonates. Limited data suggest that these may benefit some patients, but multiple cases of *Lactobacillus* bacteremia have been reported in infants and children following probiotic therapy.^228–232^
3. **Endotracheal tubes equipped with subglottic secretion drains.** (Quality of Evidence: NA).
  - Products sized for neonates are not commercially available.
4. **Silver coated endotracheal tubes. Products sized for neonates are not commercially available** (Quality of Evidence: NA).

#### Preventing VAP and/or PedVAE in pediatric patients outside the neonatal period

1. Framework for evaluating and prioritizing interventions:
  - Diagnosing VAP is as challenging in term infants and children, as it is in adults and preterm neonates. The CDC recently published definitions for pediatric ventilator-associated events, denoted PedVAE, predicated on detecting patients with sustained increases in mean airway pressure or FiO_2_ after a period of stability or improvement.^54^
  - Risk factors for VAE and VAP in infants and children are similar to those of adults.^233–237^ The majority of PedVAEs are not infection related; thus, there are additional risk factors for PedVAE beyond those for VAP alone. Neuromuscular blockade, sedative type, blood transfusions, positive fluid balance, and acute kidney injury have been associated with PedVAE.^63,69,238,239^
  - In general, most VAP prevention interventions recommended for adults are presumed to be applicable to older infants and children. Some interventions recommended for adults, however, are not available for infants and small children. For example, the smallest available endotracheal tube equipped with subglottic secretion drainage ports is size 6.0 and therefore is not an option for children under 10 years of age. Similarly, the smallest available silver-coated endotracheal tube is size 6.0.

### Essential practices for pediatric patients

The following interventions confer minimal risk of harm, and some data suggest that they may lower VAP rates, PedVAE rates, and/or duration of mechanical ventilation.

#### Avoid intubation if possible

1. **Use noninvasive positive pressure ventilation (NIPPV) or high flow oxygen by nasal cannula whenever safe and feasible** (Quality of Evidence: MODERATE).
  - Risks of NIPPV in pediatric patients mirror those for adults with the added issue that pediatric patients often need sedation to tolerate NIPPV.^240,241^
  - CPAP may be superior to high flow oxygen by nasal cannula to avoid intubation in infants with bronchiolitis.^242^

#### Minimize duration of mechanical ventilation

1. **Assess readiness to extubate daily in patients without contraindications**^243–247^ (Quality of Evidence: MODERATE).
  - Randomized controlled trials suggest that daily spontaneous breathing trials can decrease mean duration of ventilation and PICU length of stay in postoperative cardiac surgery patients.^245,248^ There is no consensus on the most effective technique for spontaneous breathing trials in pediatricpatients.^243,246^
2. **Take steps to minimize unplanned extubations and reintubations**^249,250^ (Quality of Evidence: LOW)
  - A multicenter, quality-improvement initiative tested a bundle of measures to reduce unplanned extubations.^250^ The bundle included standardized anatomic reference points and securement methods, protocols for high-risk situations, and multidisciplinary apparent-cause analyses. The bundle was associated with significant reductions in unplanned extubations and episodes of cardiovascular collapse.
3. **Avoid fluid overload** (Quality of Evidence: MODERATE).
  - Meta-analysis of the association between fluid balance and outcomes in critically ill children suggests that fluid overload is associated with increased risk for prolonged mechanical ventilation (>48 hours).^251^
  - Interventional studies on fluid management in critically ill children are sparse. One of the few available studies assessed infants at risk for acute kidney injury and fluid overload following cardiac surgery. These infants were randomized to peritoneal dialysis versus furosemide; those randomized to peritoneal dialysis were less likely to develop fluid overload and less likely to have prolonged ventilator use.^252^ The generalizability of these findings to other populations is unknown.
  - c. The Pediatric Surviving Sepsis Campaign and the Pediatric Acute Lung Injury Consensus Conference recommend limiting fluid intake, starting diuretics, and/or early renal replacement therapy for children with ARDS and for children in the postresuscitation phase of sepsis.^253,254^

#### Provide regular oral care

1. **Provide regular oral care** (Quality of Evidence: LOW).
  - Four before-and-after studies of VAP bundles that emphasized oral care reported significant decreases in VAP rates following bundle implementation.^234,255–257^
  - The American Dental Association recommends beginningoral hygiene a few days after birth in term infants. Wipe the gums with a gauze pad after each feeding to remove plaque and residual formula that could harm erupting teeth.
  - For children aged <3 years, the ADA recommends brushing children’s teeth as soon as they begin to come into the mouth using fluoride toothpaste in an amount no more than a smear the size of a grain of rice.^258^ A pea-sized amount of fluoride toothpaste is recommended for children aged 3–6 years.^258^
  - After oral hygiene, rinse and suction the mouth. Keep the oral mucosa and lips clean, moist, and intact using sponge-tipped applicators dipped in non-alcohol, non-peroxide mouth rinse.^255^

#### Elevate the head of the bed

1. **Elevate the head of the bed unless medically contraindicated** (Quality of Evidence: LOW).
  - Three before-and-after studies of VAP bundles that included head of bed elevation reported lower VAP rates.^234,256,257^
  - Many hospital cribs do not have inbuilt angle-measuring devices. Alternative measuring devices are required in these circumstances.

#### Maintain ventilator circuits

1. **Change ventilator circuits only when visibly soiled or malfunctioning or per manufacturers’ instructions** (Quality of Evidence: MODERATE).
  - A meta-analysis of 6 studies reported no difference in VAP rates or mortality with 3-day versus 7-day circuit changes.^259^ Circuit changes are therefore recommended only when the circuit is soiled or malfunctioning to minimize costs.^260,261^
  - Follow manufacturers’ instructions for use if they differ from this recommendation.
2. **Remove condensate from the ventilator circuit frequently** (Quality of Evidence: LOW).
  - Avoid draining the condensate toward the patient.^234^

#### Endotracheal tube selection and management

1. **Use cuffed endotracheal tubes** (Quality of Evidence: LOW).
  - Pediatric intensivists have historically favored uncuffed tubes due to concern that cuffs may induce subglottic stenosis in pediatric airways. Cuffing has proven safe, however, and may decrease the risk of microaspiration.^262,263^ Cuffed tubes are now recommended for term newborns and children.^264^
2. **Maintain cuff pressure and volume at the minimal occlusive settings to prevent clinically significant air leaks around the endotracheal tube, typically 20–25 cm H**_**2**_**O.**^171,262,265^ **This “minimal leak” approach is associated with lower rates of post-extubation stridor**^265^ (Quality of Evidence: LOW).
  - The potential merits of automated manometers for VAP prevention have not been studied in pediatric patients.
3. **Suction oral secretions before each position change** (Quality of Evidence: LOW).^266^

### Additional approaches to preventing VAP and VAE in pediatric patients

The following interventions are associated with minimal risks of harm and some evidence of benefit in adult patients, but data in pediatric populations are limited.

1. **Minimize sedation** (Quality of Evidence: MODERATE).
  - Daily sedative interruptions decreased duration of mechanical ventilation and ICU length of stay without increases in adverse event rates in 1 small, randomized trial.^267^
  - There is nonetheless concern that sedative interruptions will increase the frequency of unplanned extubations and reintubations in younger patients, so this practice may be safest in older pediatric patients.
2. **Use endotracheal tubes with subglottic secretion drainage ports** (Quality of Evidence: LOW).
  - This intervention has not been studied in children and is only feasible for children aged ≥10 years because the smallest available endotracheal tube with subglottic secretion drainage ports is size 6.0.
3. **Consider early tracheostomy** (Quality of Evidence: LOW).
  - A small, single-center, retrospective cohort study reported that early tracheostomy (<10 days) was associated with lower VAP rates and shorter ICU length of stay compared with late tracheostomy.^268^
  - A propensity-matched analysis of the timing of tracheostomy among children with severe traumatic brain injury reported an association between early tracheostomy and lower pneumonia rates, shorter ICU length of stay, and shorter hospital length of stay.^269^
  - A meta-analysis of retrospective cohort studies reported that early tracheostomy was associated with lower mortality rates, fewer ventilator days, and shorter ICU length of stay.^270^
  - Tracheostomy complications are more frequent in children versus adults.^271^

### Approaches that are generally not recommended for VAE and VAE prevention in pediatric patients

The following interventions have unknown impact on VAP and PedVAE rates and/or have inadequate data on risks.

1. **Prolonged systemic antimicrobial therapy for ventilatorassociated tracheitis** (Quality of Evidence: LOW)
  - One retrospective study found that prolonged antibiotics for ventilator-associated tracheitis did not protect against VAP but did increase the prevalence of multidrug-resistant organisms.^272^ Whether, when, and how long to treat ventilatorassociated tracheitis to prevent VAP in children remains unresolved.^273^
2. **Selective oropharyngeal or digestive decontamination** (Quality of Evidence: LOW)
  - A meta-analysis of 4 randomized trials in critically ill children published between 1991 and 2001 reported that selective digestive decontamination using a combination of oral and parenteral antibiotics may be associated with a decrease in pneumonia rates but no change in mortality.^274^ Trials were small, and the longterm impact on antibiotic resistance was not assessed.
3. **Prophylactic probiotics** (Quality of Evidence: LOW)
  - Probiotics should be considered with caution due to sparse data on impact in children, lack of clear benefit in adults, and case reports of *Lactobacillus* bacteremia associated with probiotic therapy in pediatric patients, including those not known to be immunocompromised.^157,163,230–232,275–278^

#### No impact on VAP rates for pediatric patients

These interventions may be indicated for reasons other than VAP prophylaxis.

1. **Oral care with chlorhexidine** (Quality of Evidence: MODERATE)
  - Chlorhexidine appears to be safe for developing teeth,^279^ but randomized controlled trials have found no difference in VAP rates, length of stay, or mortality in infants and children.^280–285^
2. **Stress-ulcer prophylaxis** (Quality of Evidence: LOW)
  - Two small studies found no impact on VAP rates.^286,287^ A larger, multicenter, cohort study and a meta-analysis reported that acid-suppressive medications were associated with higher VAP rates.^288,289^

#### Lowers VAP rates but no impact on duration of mechanical ventilation, length of stay, or mortality

1. **Silver-coated endotracheal tubes** (Quality of Evidence: LOW) a. These tubes have not been studied in children and are only feasible for children aged ≥10 years since the smallest available size is 6.0.

#### No recommendation

These interventions have limited data from pediatric studies, no impact on VAP rates or outcomes in adults, and unclear impact on costs.

1. **Closed/in-line suctioning** (Quality of Evidence: LOW)
  - Closed suctioning may be associated with fewer transient decreases in oxygenation and increases in heart rate and blood pressure compared to open suctioning, but the clinical significance of these findings is unclear.^290–292^
  - An observational study of open versus closed suctioning in children did not find any difference in VAP rates, length of stay, or mortality, but the significance of these findings are unclear given the lack of blinding and randomization.^293^

##### Recommendations to prevent NV-HAP

Little robust data exist on interventions to prevent NV-HAP. Most studies are nonrandomized, and many do not report the impact on objective outcomes such as length of stay, mortality, or antibiotic utilization. We classify potential prevention strategies into (1) practices supported by interventional studies suggesting lower NV-HAP rates, (2) practices with insufficient data of benefit or harm, and (3) practices that are not recommended, with evidence of futility or possible harm.

### Interventions that may lower NV-HAP rates with little risk of harm

#### Provide regular oral care

1. Oral care is the most commonly studied strategy to prevent NV-HAP. Before-and-after series suggest a possible benefit.^294–297^ Two large, cluster randomized trials conducted in nursing homes did not show a benefit, but their generalizability to acute-care hospitals is unknown.^298,299^ Most randomized trials in acute-care hospitals have focused on ICU patients, most of whom were on mechanical ventilation, making it difficult to discern their effect on NV-HAP.^300,301^
  - Uncertainty remains regarding the most effective protocols, including types of staff involved (eg, dentistry professionals versus nondentistry professionals), frequency of oral care, whether to include an antiseptic, and if so, what type of antiseptic to use (eg, chlorhexidine gluconate, sodium bicarbonate, hydrogen peroxide, cetylpyridinium chloride, povidone-iodine).
  - Notwithstanding the gaps in current evidence, we recommend toothbrushing daily given its benefits for oral health and the possible positive impact on objective outcomes observed in before-and-after studies in nonventilated patients and meta-analyses of randomized trials in ventilated patients.^126,127,294–297^

#### Diagnose and manage dysphagia

1. Early diagnosis and treatment of dysphagia may prevent NV-HAP, especially among neurologically impaired post-stroke patients.^302–304^
2. Potential approaches to diagnose dysphagia include nurse-administered risk assessment tools, bedside functional evaluations of swallowing, video fluoroscopic study, and fiberoptic endoscopic examination.
3. Potential options to manage dysphagia include changes in method of pill administration, adjustments in consistencies of liquids and solids, supervision or assistance with meals, use of straws, and elevation of the head of bed while eating.

#### Provide early mobilization

1. Data for early mobilization to prevent NV-HAP among hospitalized patients are sparse.^304,305^ One quasi-experiment found that bundling mobilization with other interventions reduced NV-HAP, attributable mortality, and antibiotic utilization, but the relative contribution of mobilization to these benefits is unclear.^305^ A randomized trial of engaging families to provide turning plus passive mobilization to post-stroke patients versus turning by nursing staff alone reported a significant decrease in pneumonia rates but did not report impact on length of stay or mortality.^306^ A nonrandomized controlled trial reported that mobilizing patients in 2 geriatric and respiratory wards was associated with a significant decrease in pneumonia rates compared to usual care in matched wards, although falls were significantly more frequent in the intervention group than in the control group and data were not provided on length of stay or mortality.^307^ In a quasi-experimental study, intensified postoperative physical therapy for elderly patients undergoing hip fracture surgery was associated with less pneumonia and shorter length of stay compared to historical controls.^308^
2. Additional trials are needed to better quantify the possible benefits versus fall-related harms of mobility programs. Implementation strategies are needed to increase the feasibility, frequency, and safety of mobilizing acute-care patients. In the meantime, early mobilization of patients should take into the account the risk of falls.

#### Implement multimodal interventions to prevent viral infections

1. Approximately 20%–40% of NV-HAP is attributable to viral pathogens, and the ongoing coronavirus disease 2019 (COVID-19) pandemic has highlighted the risk and morbidity of within-hospital transmission of respiratory viruses.^25,309–311^
2. Possible strategies to prevent nosocomial viral transmission include symptom screening of patients and healthcare workers, surveillance testing of all admitted patients, transmission-based precautions for patients with suspected and confirmed respiratory viral infections, universal masking when respiratory virus transmission rates are high in the hospital or in the community, assuring adequate ventilation, and vaccination of healthcare personnel and patients.^312,313^

#### Bundles

1. Multiple observational studies have reported lower NV-HAP rates after implementing prevention bundles.^305,314,315^ Effective bundles have included heterogeneous combinations of oral hygiene, bed positioning, dysphagia diagnosis and management, mobilizing patients, nasal hygiene, sedation restrictions, incentive spirometry, education for physicians and nurses, and/or electronic order-set bundles. One small randomized trial of usual care versus a bundle comprising dysphagia screening, oral care with chlorhexidine, placing the bed in the reverse Trendelenburg position, and vaccination against influenza and pneumococcus reported no difference in NV-HAP rates, length of stay, or mortality but did report a lower 1-year risk of readmission for respiratory infection.^316^
2. The respective contribution of each bundle component, the extent to which bundle components are synergistic versus additive, and the most effective combination of interventions to include in bundles remains unknown.

### Interventions with insufficient data to determine impact on NV-HAP

#### Bed positioning

1. Elevating the head of the bed is recommended to prevent VAP and VAE despite sparse evidence because some studies suggest benefit, it is simple, economical, and associated with minimal risk of harm in ventilated patients. Even fewer data, however, are available to inform whether and to what extent this applies to NV-HAP.
2. One randomized trial among critically ill patients with tetanus in Vietnam found that semirecumbent position was associated with no difference in pneumonia rates but more frequent complications including the need for tracheostomy.^317^ The generalizability of these findings to nontetanus patients in other settings is unknown.

#### Stress-ulcer prophylaxis

1. Observational studies suggest an association between stress-ulcer prophylaxis and risk for NV-HAP but we are not aware of any randomized trials assessing the impact of acid-suppressing medications on NV-HAP outside the ICU setting.^318^

### Approaches not generally recommended for routine NV-HAP prevention

#### Systemic antibiotic prophylaxis

1. Randomized trials of prophylactic antibiotics in acute stroke patients show no impact on pneumonia rates, functional outcome, or mortality.^319^

## Section 5: Performance measures

### Monitoring and reporting

1. Regular monitoring and internal reporting of patient outcomesand adherence rates to recommended prevention strategies (“process measures”) are important quality improvement strategies.
2. Both outcome and process-measure reporting are likely beneficial: improving outcomes is the primary goal of care improvement programs but process of care surveillance can help identify specific processes to target for improvement.
3. Report outcome measures to key organizational stakeholders including frontline care providers, service leaders (medical, nursing, respiratory therapy), and senior hospital administrators. Reporting these data back to providers and leaders has been associated with improvements in both performance rates and outcomes.^320–325^
4. Report process measures internally only. External reporting of process measure data is not appropriate given substantial variability in the ways different organizations define, collect, analyze, and present process measure data.

### Process measures

1. Process-measure definitions and measurement strategies vary widely.
2. For organizations that collect and report process measures:
  - Clearly define measures including data sources, inclusion and exclusion criteria, frequency of monitoring, and numerator and denominator criteria.
  - Develop a formal system to document compliance.
    - Compliance can be measured via direct observationsor via audits of patient charts, bedside checklists, and/or electronic medical records. Periodically validate the accuracy of paper and/or electronic documentation.
  - Perform assessments regularly.
    - The optimal frequency of assessments (eg, once daily, twice daily, or weekly) is not known but can likely be adjusted based on compliance rates and unit size.
    - An analysis of a large collaborative quality improvement effort suggests that the following approach can be used to determine the frequency of process measure assessments.^326^
  - Start by measuring processes daily. If compliance is consistently high for a given process, then decrease the frequency of measures (ie, once every 2–3 days or once per week, and if compliance continues to be high, then decrease to once per month). If compliance is low or variable, then continue with daily measurements.
  - For units with at least 30 ventilator days per month, measuring compliance on 7 consecutive days per month provides accurate performance estimates
  - For units with <30 ventilator days per month, daily data collection is required to achieve accurate performance estimates.
  - There is no consensus on how best to define adherence to different process measures and definitions for measuring adherence vary widely.
    - Several published studies provide examples of how process measures were defined.^118,325,327–329^ These approaches can be used as starting points to come up with local strategies to define adherence.

### Prevention bundles

1. Prevention bundles are widespread in critical care and have been associated with decreases in VAP, VAE, NV-HAP, and in some cases, length of stay and mortality.
2. A meta-analysis of 13 observational studies in adults found that implementation of ventilator bundles was associated with a 10% decrease in mortality, less time to extubation, and shorter length of stay.^330^ All the studies included in this analysis, however, were before-and-after or time-series analyses. It is therefore difficult to discern the extent to which lower VAP rates and better outcomes were due to prevention bundles versus changes in patient mix or unrelated changes in care.^331^ Observational analyses of pediatric bundles have reported significant decreases in VAP rates, but few data are available on other outcomes.^332^
3. Prevention bundles have only been tested in 1 randomized trial. Researchers in Brazil randomized 188 ICUs to a multifaceted intervention including checklists for the prevention of VAP, daily goal assessments, and clinician prompts. There were no significant differences between intervention versus control units in VAP rates or mortality rates.^329^ These results should be interpreted with caution, however, given that baseline adherence with some process measures was already high (eg, head of bed elevation), adherence with other measures did not improve (eg, light sedation, low tidal volume ventilation), and the intervention period may have been too brief to achieve significant changes.^333^
4. There is no consensus on which processes to include in VAP/VAE prevention bundles. Bundles vary widely among different centers.^330,334^
5. Ventilator bundle components potentially associated with lower mortality rates include staff education, performance feedback, and in adults, elevating the head of the bed, minimizing sedation, and assessing readiness for extubation.^110,113,330,335^ Additional promising strategies include conservative fluid management, low tidal volume ventilation, and early mobility.^77,119,336^
6. Compliance can be reported for each process measure individually and/or as ‘all or none’ compliance with a bundle of process measures. For ‘all or none’ compliance, credit is given only if all components have been accomplished and documented; if any components were not performed and/or were not documented, no credit is given.

### Outcome measures

1. Conduct surveillance for all VAEs including VAC, IVAC, and PVAP in adult ICUs.^54,337^ Report PedVAE rates in pediatric and neonatal ICUs. Report location-stratified rates for all events included in VAE and PedVAE algorithms. Report standardized infection ratios (SIRs) for all events included in VAE algorithms.^54^1. VAE and PedVAE incidence density.
  - Numerator: total number of VAEs (including VAC+IVAC+PVAP) or PedVAEs
  - Denominator: total ventilator days
  - Multiply by 1,000 and express as VAEs or PedVAEs per 1,000 ventilator days
2. IVAC incidence density (adults)
  - Numerator: total number of IVACs and PVAPs
  - Denominator: total ventilator days
  - Multiply by 1,000 and express as the IVAC rate per 1,000 ventilator days
3. PVAP incidence density (adults)
  - PVAP rate
  - Numerator: total number of PVAPs
  - Denominator: total ventilator daysiv. Multiply by 1,000 and express as the overall PVAP rate per 1,000 ventilator days
4. Total VAE SIR (adults)
  - Numerator: total number of VAEs, including VAC, IVAC, and PVAP
  - Denominator: total number of predicted VAEs
5. IVAC plus SIR (adults)
  - Numerator: total number of IVACs (including PVAPs)
  - Denominator: total number of predicted IVACs (including PVAPs)
6. Note that the combined outcome of IVAC including PVAPsis sometimes referred to as “IVAC-plus.”

### External reporting

1. VAE is a potentially appropriate metric for public reporting, interfacility comparison, and pay-for-performance programs. Better data on the responsiveness of VAE to quality-improvement programs are necessary, however, before recommending VAEs for interfacility comparisons or pay-for-performance programs. Suitable risk adjustment strategies are also needed.
2. PVAP is not suitable for external reporting because substantial variability in clinical and laboratory practices in the acquisition, processing, and interpretation of culture data preclude meaningful comparisons of PVAP rates between institutions.
3. VAP rates generated using former NHSN surveillance definitions are not appropriate for external reporting in light of their considerable subjectivity.
4. Hospitals in states that have mandatory reporting laws must collect and report data as required by their state. Pennsylvania and South Carolina are currently the only 2 states that require hospitals to report VAEs. Local and state health departments can provide specific information on public reporting requirements.

## Section 6: Implementation of VAP, VAE, and NV-HAP prevention strategies

Prevention of VAP, VAE, and NV-HAP requires implementing best practices to reduce the risk of infection and nurturing a culture that supports implementation. Accountability is an essential principle for preventing healthcare-associated infections. It provides the necessary translational link between science and implementation. Without clear accountability, scientifically based implementation strategies will be used in an inconsistent and fragmented way, decreasing their effectiveness in preventing HAIs. Accountability begins with the chief executive officer and other senior leaders who must make preventing healthcare-associated infections an organizational priority. Senior leadership is accountable for providing adequate resources for effective implementation of a healthcare-associated infection prevention program. These resources include necessary personnel (clinical and nonclinical), education, and equipment.

Engagement, education, execution, and evaluation are common attributes of successful care improvement programs.^321,338^ These attributes are elaborated below.

### Engage

#### Develop a multidisciplinary team

1. Multidisciplinary teams set goals, define each step in the implementation process, and monitor progress in reaching goals.^79,83,234,339–341^ Programs developed by team consensus are more effective and increase guideline adherence.^234,339,342^ Multidisciplinary teams include representatives from all disciplines that care for ventilated patients, including, at a minimum, unit directors, physicians, nurses, and respiratory therapists. Other partners that can strengthen the team include infection preventionists, pharmacists, nutritionists, physical therapists, occupational therapists, family members, and patient advocates.^77,83,325,343–347^

#### Involve local champions

1. Identify local champions, including formal (eg, medical director, nursing director, charge nurses, director of respiratory therapy) and informal leaders (eg, engaged frontline staff).^321,325,328,340,343–345,348,349^
2. Local champions are important to success because they engage stakeholders, educate peers, encourage ongoing improvement, and increase buy-in and ownership by both staff and administrators.^234,321,325,342,346,348,350^
3. Local champions should know their hospital’s interests and needs and should be able to shape strategies to match local unit culture, monitor progress, and facilitate necessary changes during implementation.^320,351–353^ Early and continual communication between local champions and frontline staff allows providers to ask questions, resolve concerns, prepare for action, and sustain improvements.^320,350,352^

#### Utilize peer networks

1. Horizontal networking of peers across hospitals can promote and increase compliance with evidence-based best practices. Voluntary peer networks encourage collaboration, analysis of performance, accountability, and commitment to specific goals.^77,79,325,327,345,354,355^ Comparing progress and benchmarks between ICUs can help units better understand their local strengths and weaknesses, learn from best practices, brainstorm solutions to common problems, and promulgate local successes.^77,79,327,336,345,353^

### Educate

#### Provide education sessions

1. The introduction of evidence-based practices in the clinical setting should be supported by active and multifaceted education programs.^77,330,356^
2. Education sessions help summarize evidence, explain new processes, set expectations, and encourage staff to adopt recommended practices.^320,327,357,358^
3. Education sessions can include workshops, hands-on training, conferences, slide presentations, and/or interactive discussions; employing multiple teaching modalities can help meet diverse learning styles.^327,341,350,359,360^ Both local champions and topic experts (eg, infection preventionists) can lead staff education.^234,351,355,361^
4. Education sessions must be informative and relevant for the learner; therefore it is important to have multidisciplinary educational programs customized for different levels of training and different specialties.^81,321,322,339^
5. Ongoing staff education helps maintain high levels of compliance with recommended practices.^323,345,347,359,362^
6. Implementation of experiential learning strategies (simulation models, play activities, knowledge and attitude competencies, role-playing and feedback) may improve bundle adherence.^341^
7. Educating patients and family members may help them better engage with and support the medical team’s plan of care.^336,363^

#### Provide educational materials

1. Provide educational materials to staff that summarize the evidence, support self-study, and remind staff about new practices.^364^
2. Examples of educational materials include smartphone applications, interactive websites, pocket cards, brochures, posters, fact sheets, daily guides, guideline summaries, flow sheets and 1-page bulletins.^234,323,325,345,362–366^

### Execute

#### Standardize care processes

1. Standardize care processes through the implementation of guidelines, bundles, protocols or pathways. Standardization helps establish new care processes as “normal behaviors” for staff.^77,79,81,83,320,347,349,353,358^
2. Interventions to improve adherence with best practices in the ICU that include protocols with or without education are associated with the greatest improvements in processes of critical care.^367^
3. Daily multidisciplinary rounds are widely recommended.^368,369^ Rounds should follow a structured format and include discussion about the patients’ goals for the day, consideration of what resources and actions are necessary to achieve these goals, and identification of potential barriers and/or safety issues.^322,325,344,363,370,371^

#### Create redundancy

1. Build redundancy or independent checks into care delivery processes to increase the likelihood that best practices are followed.^320,321,347,363,367,372^
2. Redundancy includes reminders about best practice and can take the form of posters, bulletins, pens, stamps, pocket cards, 1-page signs, daily goal lists in patient rooms, checklists, and preprinted order sets, text messages, and screensavers on clinical computers.^81,321,323,351,355,366,372–378^
3. Engage family members to assist with preventive care as appropriate and/or to discuss prevention practices with the care team daily. This provides an external prompt for the performance of best practices and can help increase patient acceptance of practices such as oral care, mobilization, and delirium prevention.^325,363^
4. The combination of education and reminders significantly improves process of care performance rates.^357^

### Evaluate

#### Measure performance

1. Measure performance using frequent formal and informal audits of clinical practice.^77,81,83,321,327,363,379^
2. Measuring process and outcome measures enhances awareness, establishes expectations, creates urgency, and rewards changes in behavior.^320,360–362^
3. Evaluating performance provides an ongoing, real-time image of actual implementation rates. Areas of poor compliance can be rapidly identified and rectified.^321,341,346,351,358,374,379,380^ If compliance remains poor in one area, the improvement team should walk the process with staff to gain additional insights into barriers to implementation.^321,341,346,351,352^
4. Analyze all or a representative sample of VAEs for etiology and preventability. Pneumonia, pulmonary edema, acute respiratory distress syndrome, and atelectasis are the precipitants for most VAEs.^17,19,22,58,60,61^ Use these analyses to select and refine prevention strategies that address the most frequent and preventable causes of VAEs in the specific unit of interest.^83,351^

#### Provide feedback to staff

1. Provide regular feedback on process and/or outcome data to staff.^77,79,320,322,355,360,372,375^ Feedback can be provided via dashboards, wall displays, or during meetings.^81,234,320,351,358,364,365^
2. Providing feedback helps staff appreciate how their efforts to improve are affecting performance rates and patients’ outcomes. This helps maintain staff motivation and can boost adherence to new processes.^79,321,327,351,381^
3. Feedback is also important for future efforts because feedback helps pinpoint new areas for improvement and marks successful transitions to new standards of care.^320,321,351,379,381^

### Barriers and facilitators for adoption

Qualitative studies have identified several common barriers and facilitators to the implementation of VAE/VAP prevention programs.

1. Barriers related to staff workload and time (ie, competing priorities, data collection burden, not enough time), staff turnover, and lack of leadership support can impede implementation progress.^382,383^
2. Barriers may also include a unit culture that does not prioritize preventive care and lack of a structured interdisciplinary approach to minimizing sedation and facilitating liberation from mechanical ventilation.^378^
3. Facilitators for bundle adherence include ‘reflective motivation’ (ie, VAE and VAP are perceived as serious and common problems among ICU patients; providers consider prevention measures useful to lower VAE or VAP rates and improve patients’ outcomes). Reflective motivation can be achieved through increasing knowledge and understanding or through eliciting positive (or negative) feelings about adopting new practices.^383^
4. Goddard et al,^384^ using a Theoretical Domains Framework of behavior change, found that the social influences domain (local champions, ICU leadership, engagement between providers and family members) and behavioral regulation domain (feedback and having a unit protocol) may act as barriers or facilitators to early rehabilitation.

### Positive examples and resources

Many successful improvement programs have been published and provide additional insights into practical strategies to engage multidisciplinary teams, educate key stakeholders, implement multifaceted interventions, and achieve significant reductions in VAE, VAP, and/or NV-HAP.^77,83,305,325,341,345,358,367^ In addition, The Society of Critical Care Medicine’s (SCCM) ICU Liberation initiative offers numerous tools and resources to assist hospitals with implementing bundles designed to improve patient outcomes and reduce the risk of long-term consequences of critical illness.^369,385^

## Figures and Tables

**Table 1.** Quality of Evidence^a^

Category	Definition
HIGH	Highly confident that the true effect lies close to that of the estimated size and direction of the effect. Evidence is rated as “HIGH” quality when there are a wide range of studies with no major limitations, there is little variation between studies, and the summary estimate has a narrow confidence interval.
MODERATE	The true effect is likely to be close to the estimated size and direction of the effect, but there is a possibility that it is substantially different. Evidence is rated as “MODERATE” quality when there are only a few studies and some have limitations but not major flaws, there is some variation between studies, or the confidence interval of the summary estimate is wide.
LOW	The true effect may be substantially different from the estimated size and direction of the effect. Evidence is rated as “LOW” quality when supporting studies have major flaws, there is important variation between studies, the confidence interval of the summary estimate is very wide, or there are no rigorous studies.

a Based on the CDC Healthcare Infection Control Practices Advisory Committee (HICPAC) “Update to the Centers for Disease Control and Prevention and the Healthcare Infection Control Practices Advisory Committee Recommendations Categorization Scheme for Infection Control and Prevention Guideline Recommendations” (October 2019), the Grades of Recommendation, Assessment, Development, and Evaluation (GRADE),^386^ and the Canadian Task Force on Preventive Health Care.^387^

**Table 2.** Summary of Recommendations to Prevent VAP and/or VAE in Adult Patients

Category	Rationale	Intervention	Quality of Evidence
Essential practices	Good evidence that the intervention decreases the average duration of mechanical ventilation, length of stay, mortality, and /or costs. Benefits likely outweigh risks.	Avoid intubation and prevent reintubation • Use high-flow nasal oxygen or noninvasive positive pressure ventilation (NIPPV) as appropriate whenever safe and feasible^91–93,96,99^	HIGH
		Minimize sedation^105,106^ • Avoid benzodiazepines in favor of other agents^106^ • Use a protocol to minimize sedation^110^ • Implement a ventilator liberation protocol^113^	MODERATE
		Maintain and improve physical conditioning^113,120–123^	MODERATE
		Elevate the head of the bed to 30–45°^125,388–390^	LOW^a^
		Provide oral care with toothbrushing but *without* chlorhexidine^126,127^	MODERATE
		Provide early enteral vs. parenteral nutrition^131^	HIGH
		Change the ventilator circuit only if visibly soiled or malfunctioning (or per manufacturers’ instructions)^391–394^	HIGH
Additional approaches	Good evidence that the intervention improves outcomes in some populations, but may confer some risk in others.	Use selective oral or digestive decontamination in countries and ICUs with low prevalence of antibiotic-resistant organisms^128,134,135^	HIGH^a^
	May lower VAP rates but insufficient data to determine impact on duration of mechanical ventilation, length of stay, or mortality.	Utilize endotracheal tubes with subglottic secretion drainage ports for patients expected to require >48–72 hours of mechanical ventilation^395^	MODERATE
		Consider early tracheostomy^144^	MODERATE
		Consider postpyloric rather than gastric feeding for patients with gastric intolerance or at high risk for aspiration^131,147^	MODERATE
Generally not recommended	Inconsistently associated with lower VAP rates and no impact or negative impact on duration of mechanical ventilation, length of stay, or mortality.	Oral care with chlorhexidine^75,128–130,150^	MODERATE
		Probiotics^153–156^	MODERATE
		Ultrathin polyurethane endotracheal tube cuffs^165–167^	MODERATE
		Tapered endotracheal tube cuffs^169^	MODERATE
		Automated control of endotracheal tube cuff pressure^170,171,174,175^	MODERATE
		Frequent cuff-pressure monitoring^176^	MODERATE
		Silver-coated endotracheal tubes^178^	MODERATE
		Kinetic beds^180^	MODERATE
		Prone positioning^181,183,a^	MODERATE
		Chlorhexidine bathing^184–186,a^	MODERATE
	No impact on VAP rates, average duration of mechanical ventilation, length of stay, or mortality.^a^	Stress-ulcer prophylaxis^190,191,193^	MODERATE
		Monitoring residual gastric volumes^194^	MODERATE
		Early parenteral nutrition^195^	MODERATE
No recommendation	No impact on VAP rates or other patient outcomes, unclear impact on costs.	Closed endotracheal suctioning systems^197–199^	MODERATE

Note. VAP, ventilator-associated pneumonia.

a May be indicated for reasons other than VAP prevention.

**Table 3.** Summary of Recommendations to Prevent VAP and/or VAE in Preterm Neonates

Category	Rationale	Intervention	Quality of Evidence
Essential practices	May lower VAP and/or PedVAE rates and have minimal risks of harm. Benefits likely outweigh potential risks.	Use non-invasive positive pressure ventilation in selected populations^62,205,206^	HIGH
		Minimize the duration of mechanical ventilation	HIGH
		Use caffeine therapy to facilitate extubation^396,397^	HIGH
		Assess readiness to extubate daily	LOW
		Manage patients without sedation whenever possible^209,210^	LOW
		Avoid unplanned extubations and reintubations^212^	LOW
		Avoid reintubation by using nasal CPAP, non-invasive positive pressure ventilation (NIPPV), of high flow nasal cannula in the post-extubation period^396^	HIGH
		Provide regular oral care with sterile water	LOW
		Change the ventilator circuit only if visibly soiled or malfunctioning^259^ (or per manufacturer’s instructions)	LOW
Additional approaches	Unknown impact on VAP and VAE rates but risk of harm likely minimal. Reasonable to consider implementing if rates remain elevated despite essential practices.	Lateral recumbent positioning^215^	LOW
		Reverse Trendelenberg positioning	LOW
		Closed/in-line suctioning systems^216,217^	LOW
		Oral care with maternal colostrum^218^	MODERATE
Generally not recommended	Unknown impact on VAP rates and inadequate data on risks.	Regular oral care with an antiseptic or Biotene^219^	LOW
	May be harmful. Risk-benefit balance does not favor intervention, unless specifically indicated for reasons other than VAP prevention	Histamine-2 receptor antagonists^220,221^	MODERATE
		Prophylactic broad-spectrum antibiotics^222–225^	MODERATE
		Daily spontaneous breathing trials^398,399^	LOW
		Daily sedative interruptions	LOW
		Prophylactic probiotics or synbiotics^228,229^	LOW
	Not recommended because appropriate products are not available or approved for use in this population.	Endotracheal tubes with subglottic secretion drainage ports	NA
		Silver-coated endotracheal tubes	NA

Note. CPAP, continuous positive airway pressure; VAP, ventilator-associated pneumonia.

**Table 4.** Summary of Recommendations to Prevent VAP and/or PedVAE in Pediatric Patients

Category	Rationale	Intervention	Quality of Evidence
Essential practices	Interventions with minimal risk of harm and some data that they may lower VAP rates, PedVAE rates, and/or duration of mechanical ventilation.	Avoid intubation if possible. Use non-invasive positive pressure ventilation for selected populations^240–242^	MODERATE
		Assess readiness to extubate daily in patients without contraindications^244–248^	MODERATE
		Take steps to minimize unplanned extubations and reintubations^249^	LOW
		Avoid fluid overload^251,253,254^	MODERATE
		Provide regular oral care (i.e., toothbrushing or gauze if no teeth)^234,256,257^	LOW
		Elevate the head of the bed unless medically contraindicated^234^	LOW
		Change ventilator circuits only if visibly soiled or malfunctioning^259^ (or per manufacturer’s instructions)	MODERATE
		Prevent condensate from reaching the patient^234,266^	LOW
		Use cuffed endotracheal tubes^262–264^	LOW
		Maintain cuff pressure and volume at the minimal occlusive settings	LOW
		Suction oral secretions before each position change	LOW
Additional approaches	Risk of harm likely minimal with some evidence of benefit in adult patients, but data in pediatric populations are limited. Reasonable to consider implementing if rates remain elevated despite essential practices.	Interrupt sedation daily^267^	MODERATE
		Utilize endotracheal tubes with subglottic secretion drainage ports for older pediatric patients expected to require >48 or 72 hours of mechanical ventilation^395^	LOW
		Consider early tracheostomy^268–270^	LOW
Generally not recommended	Unknown impact on VAP rates and inadequate data on risks.	Prolonged systemic antimicrobial therapy for ventilator-associated tracheitis^272^	LOW
		Selective oropharyngeal or digestive decontamination^274^	LOW
		Prophylactic probiotics^163^	LOW
	No impact on VAP rates.^a^	Oral care with antiseptics such as chlorhexidine^280,284,285^	MODERATE
		Stress-ulcer prophylaxis^286–288^	LOW
	Lowers VAP rates in adults but no impact on duration of mechanical ventilation, length of stay, or mortality.	Silver-coated endotracheal tubes	LOW
No recommendation	Limited data on pediatric patients, no impact on VAP rates or outcomes in adults, unclear impact on costs	Closed or in-line suctioning^293^	LOW

Note.VAP, ventilator-associated pneumonia

a May be indicated for reasons other than VAP prevention.
